# Impact of the 2016 Kumamoto earthquake on patients with nephrogenic diabetes insipidus and preparations for the future

**DOI:** 10.1002/ccr3.3718

**Published:** 2020-12-30

**Authors:** Hiroshi Tamura, Hiroko Nagata, Keishiro Furuie, Shohei Kuraoka, Yuko Hidaka, Hitoshi Nakazato

**Affiliations:** ^1^ Department of Pediatrics Faculty of Life Sciences Kumamoto University Kumamoto Japan

**Keywords:** children, disasters, Nephrogenic diabetes insipidus, water supply

## Abstract

Patients with nephrogenic diabetes insipidus should establish a support network system by contacting the government to ensure that water can be preferentially obtained in the event of a disaster and create and carry a medical alert card.

## Graphical abstract

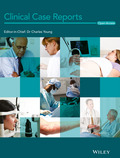

## 1 BACKGROUND

Nephrogenic diabetes insipidus (NDI) is characterized by the decreased ability of the kidney to remove water from urine. It is caused by resistance of the kidney to arginine vasopressin, also called antidiuretic hormone (ADH), and the clinical manifestations include excessive thirst (polydipsia) and excretion of a large amount of dilute urine (polyuria). In adults, this condition can be caused by numerous factors such as lithium use; however, in children, the cause of the disease is typically hereditary. The most common genetic cause is an X‐linked mutation in the arginine vasopressin receptor type 2 (AVPR2) gene; rarely, the cause can be an autosomal recessive or dominant mutation in the aquaporin 2 gene.^1^, ^2^ In patients with NDI, water intake is essential, because reduced water intake causes hypernatremia and impaired consciousness. If low water intake continues for a long period of time, it can lead to growth disorders and mental retardation.

Japan is a country often affected by major natural disasters. According to the Japan Meteorological Agency, there is a 70%‐80% probability that a major earthquake with the Nankai Trough as the epicenter will occur within 30 years. The Great Hanshin Awaji Earthquake disaster in 1995 proved the importance of patients undergoing peritoneal dialysis receiving a supply of clean water, electricity, a clean environment, and a network linking dialysis hospitals.^3^ On the other hand, there is little awareness that free water intake is essential for patients with NDI during a disaster.

We report cases of patients with NDI experiencing water shortage following a series of Kumamoto earthquakes in 2016, with the aim of being prepared for a future disaster.

## 2 CASE PRESENTATION

During the Kumamoto earthquake, a total of 6 children with NDI were being managed by pediatricians in the Kumamoto Prefecture. All the cases were boys (age range: 2‐16 years), including two pairs of brothers (Table 1). All patients were residents of Kumamoto city. Of the 6 cases, 5 had an *AVPR2* gene mutation, whereas the 1 remaining case was suspected to have a gene abnormality. Further, 5 cases were being treated with hydrochlorothiazide and 4 with spironolactone. At the time of the study, Case 5 was not administered either of these medications owing to previous poor compliance. All cases stored water at home, and the quantity of water ranged from several plastic bottles to 4 days’ worth of water.

**TABLE 1:** Summary of children with congenital nephrogenic diabetes insipidus in Kumamot

Case	1	2 (Brother of Case 1)	3	4 (Brother of Case 3)	5	6
Age/sex	14 y/male	7 y/male	15 y /male	12 y /male	16 y /male	2 y /male
Gene abnormality	*AVPR2*	*AVPR2*	*AVPR2*	*AVPR2*	*AVPR2*	AVPR2 suspected^a^
Treatment	Hydrochlorothiazide Spironolactone	Hydrochlorothiazide Spironolactone	Hydrochlorothiazide Spironolactone	Hydrochlorothiazide Spironolactone	none	Hydrochlorothiazide
Water stock before the earthquake	3 d ( plastic bottles + Bath water)	3 d (plastic bottles + Bath water)	Several plastic bottles	Several plastic bottles	1‐2 d ( plastic bottles)	3‐4 d (plastic bottles)
home	half destruction	half destruction	intact	no problem intact	intact	intact
Water outage	10 d	10 d	2 d	2 d	10 d	A one day
Water stock after the earthquake	Evacuation shelter	Evacuation shelter	Homecoming home	Homecoming home	Well water at home	plastic bottles

^a^ The grandfather of case 6 has abnormal AVPR2 gene.

During the earthquake, the house of Cases 1 and 2 was partially destroyed, whereas the houses of the remaining cases were unaffected. The water outage lasted for 1‐10 days. Cases 1 and 2 shifted to an evacuation center, and Cases 3 and 4 returned to their mother's home located outside Kumamoto city and had sufficient water supply. Case 5 lived at home and used well water. Case 6, a toddler, required less water and had sufficient water stored in plastic bottles to last until water supply resumed.

Cases 1 and 2 are described in further detail ahead.

Case 1 was a junior high school boy. Approximately 1 month after his birth, poor weight gain was observed. When he visited a physician due to a head injury at 8 months, it was found that the patient experienced hypernatremia, and he was diagnosed with NDI following close examination. To reduce his urine output, oral hydrochlorothiazide and spironolactone administration, salt restriction, and regular urination were applied. These treatments have been continued in outpatient care since the diagnosis.

Case 2 was an elementary school boy who was the brother of Case 1. A genetic analysis of Case 1 was not been performed when Case 2 was born. Because Case 2 was a boy, he was referred to a physician for screening for NDI 16 days after birth and hypernatremia was observed, which strongly indicated NDI. The patient was initiated on hydrochlorothiazide, and spironolactone was added later. These treatments were then continued in outpatient care.

With the approval of the cases and their parents, genetic analysis was performed, indicating that the cases were hemizygous for the *AVPR2* missense mutation (p.Ser126Phe). The mother was identified to be a heterozygote of the gene abnormality.

Cases 1 and 2 produced approximately 9 and 5 L of urine daily, respectively. In preparation for a potential disaster, they stored 24 L water in plastic bottles and approximately 40 L tap water in a bath at home. They had shifted five times within the Kyushu area every few years because of their father's employment. Before the occurrence of the earthquake, they had just shifted from a nearby prefecture to Kumamoto city in early April. Although they had appointment scheduled at our hospital, it was due for a date after the earthquake.

At the time of the foreshock on 14 April 2016, there were no major damages to the house or lifeline utilities of Cases 1 and 2. However, at the time of the main shock on April 16, their house was partially destroyed and the water supply was discontinued. They called our hospital for assistance. Because the hospital has a seismic isolation structure, major damage had been avoided. However, the hospital was accepting inpatients from two other public hospitals in Kumamoto city that were unable to continue hospitalization and was thus in a state of mayhem. The on‐duty doctor encouraged the patients to purchase water themselves. Accordingly, they approached a convenience store but found that water was sold out; thereafter, they went to nearby prefectural high school, but it was not open because it was not an evacuation center in Kumamoto city. They contacted the Kumamoto City Hall to enquire about water availability but were informed that it was not available. Subsequently, they approached another evacuation center and were informed that they could not secure water.

A Kumamoto city employee who noticed the situation then learned of their illness and contacted the Waterworks Bureau, who prioritized these patients for receipt of water while ensuring that other individuals remained unaware about it. The supply began on April 17, and water was delivered to them in plastic bottles. The Family Association for NDI in Japan then contacted Cases 1 and 2 to offer support but only after they had already secured water. The two cases stayed at an evacuation center until May, and a decrease in renal function was not observed.

## 3 DISCUSSION

We examined 6 pediatric cases of NDI residing in Kumamoto city, which was severely damaged by the earthquake. Cases 1 and 2 had only recently shifted to Kumamoto city when the earthquake occurred. Our hospital was not completely aware about the patients, and their family had no knowledge of nearby places to secure water. The Kumamoto earthquake reaffirmed the importance of being connected to various groups and their members.

Although there was no injury or dehydration for patients with NDI during the Kumamoto earthquake, these patients require special attention when they are injured in the event of a disaster and they may need urgent treatments or surgery. If a solution for correcting extracellular fluid is administered during the fasting period of surgery, hypernatremia can easily progress and central nervous symptoms, such as impaired consciousness and convulsions, may occur. In some cases, there may be neurological sequelae. Cases where patients have died owing to the difficulty of adjusting the serum sodium concentration during surgery have been reported.^4^

Cases wherein NDI is indicated on admission due to trauma or emergency surgery are rare. In emergency situations, it is typically difficult to elucidate the patient's medical history due to impaired consciousness. Neurogenic diabetes insipidus could be predicted as a complication of head trauma or central nervous system disease; however, if a patient with NDI refers with a chief complaint unrelated to this, it is difficult to consider NDI as a cause of polyuria. Although NDI is a rare disease, it is likely that clinicians will encounter it in routine practice. It is estimated that there are approximately 400 patients with congenital NDI in Japan.^4^ In the past, these patients often experienced neurological sequelae such as mental retardation due to dehydration and hypernatremia during infancy. However, in recent years, advances in diagnosis have led to an increase in the number of cases living a normal life without any issues. This indicates that there is an increase in the opportunities for nonspecialist clinicians to treat patients with NDI.^5^ To notice NDI in emergency situations and to avoid serious complications, it is necessary to distinguish diabetes insipidus from osmotic diuresis, wherein polyuria is present and serum sodium concentration tends to increase.

In neurogenic diabetes insipidus, ADH administration controls urine output; however, in NDI, this control can only be achieved by compensating for the loss of free water from urine by infusion. Typically, when there is a high volume of urine and a tendency to dehydrate, rehydration with extracellular fluid is likely to be initiated, particularly in postoperative conditions. As described above, this promotes hypernatremia. Frequent monitoring of urine volume, serum sodium levels, urinary sodium level, urinary potassium level, and circulating blood volume is necessary for proper infusion management in patients with NDI. A large amount of free water with a poor electrolyte content is excreted from urine in NDI. Therefore, a published method states the use of a 5% glucose solution or a mixture of 5% glucose solution and saline solution (3:1).^6^ Resuming the consumption of drinking water at the earliest based on the patient's needs facilitates management.^6^, ^7^ If hypernatremia is already present, remedial measures should be slowly performed to avoid the worsening of neurological symptoms.

Diabetes insipidus is not a common disease, and NDI is particularly rare; nevertheless, it is a disease that can lead to serious consequences if the initial response is incorrect. In certain situations, it is difficult to understand a patient's medical history, and clinicians may be compelled to treat patients with grave conditions. Therefore, it is important to establish a system similar to the card system used by patients with diabetes.

## 4 CONCLUSIONS

We investigated the effects of the Kumamoto earthquake on 6 patients with NDI managed by pediatricians in the Kumamoto Prefecture and identified that it is important for patients with NDI to establish lines of communication, supplies, and a support network system in the event of a disaster. A support network system can be established by contacting the government in advance, including the liaison for childhood and perinatal care at the time of a disaster, to ensure that water is preferentially obtained in the event of a disaster. Furthermore, it may be necessary that the patients create and carry a medical alert card for NDI that provides information regarding the necessity of water and includes contact information of guardians and doctors.

We believe this experience will help children with NDI during the time of a disaster in the future.

## CONFLICT OF INTEREST

The authors have declared that no conflict of interest exists.

## AUTHOR CONTRIBUTIONS

HT: cared for the patients and designed the project. HN, KF, YH, and SK: collected clinical information. HN supervised this study. All authors have read and accepted the manuscript.

## INFORMED CONSENT

Informed consent was obtained from all individual participants included in the study.
